# Trends in estrogen and progesterone receptors in prostate cancer: a bibliometric analysis

**DOI:** 10.3389/fonc.2023.1111296

**Published:** 2023-06-09

**Authors:** Wenqiang Liao, Xuxia Sui, Gaoming Hou, Mei Yang, Yuxue Lin, Junjie Lu, Qingtao Yang

**Affiliations:** ^1^ Department of Urology, Second Affiliated Hospital of Shantou University Medical College, Shantou, China; ^2^ Laboratory of Pathogenic Biology, Shantou University Medical College, Shantou, China; ^3^ Department of Clinical Medicine, Shantou University Medical College, Shantou, China

**Keywords:** prostate cancer, estrogen receptor, progesterone receptor, bibliometric analysis, Citespace, VOSviewer, Bibliometrix

## Abstract

**Introduction:**

The bibliometric analysis aims to identify research trends in estrogen receptor (ERs) and progesterone receptor (PRs) in prostate cancer (PCa), and also discuss the hotspots and directions of this field.

**Methods:**

835 publications were sourced from the Web of Science database (WOS) from 2003 to 2022. Citespace, VOSviewer, and Bibliometrix were used for the bibliometric analysis.

**Results:**

The number of published publications increased in early years, but declined in the last 5 years. The United States was the leading country in citations, publications, and top institutions. Prostate and Karolinska Institutet were the most publications of journal and institution, respectively. Jan-Ake Gustafsson was the most influential author based on the number of citations/publications. The most cited paper was “Estrogen receptors and human disease” by Deroo BJ, published in the Journal of Clinical Investigation. The most frequently used keywords were PCa (n = 499), gene-expression (n = 291), androgen receptor (AR) (n = 263), and ER (n = 341), while ERb (n = 219) and ERa (n = 215) further emphasized the importance of ER.

**Conclusions:**

This study provides useful guidance that ERa antagonists, ERb agonists, and the combination of estrogen with androgen deprivation therapy (ADT) will potentially serve as a new treatment strategy for PCa. Another interesting topic is relationships between PCa and the function and mechanism of action of PRs subtypes. The outcome will assist scholars in gaining a comprehensive understanding of the current status and trends in the field, and provide inspiration for future research.

## 1 Introduction

Prostate cancer (PCa) is a complex problem for middle-aged men in modern society. Based on worldwide cancer data in 2020, there were 1,414,259 new cases and 375,304 deaths worldwide (3.8% of all deaths due to cancer in men), making it the second-most prevalent cancer in men (behind lung cancer), and the fifth leading cause of cancer death (1). In over half of the world’s countries (112 out of 185 countries), PCa is the most commonly diagnosed cancer among men. It is the primary driver of cancer deaths among men in 48 countries, with the highest mortality rates in backward regions, such as the Caribbean, sub-Saharan Africa, and Micronesia (1). The pivotal role of androgen receptor (AR) and its variants have been used as potential predictive and prognostic biomarkers in several stages of PCa (2, 3). The pioneering work of Professor Charles Huggins in 1941 demonstrated androgen deprivation therapy (ADT). ADT is the basic treatment option for locally progressive PCa and castration-resistant prostate cancer (CRPC), and is the basis for a variety of novel combination therapy options, according to the European Association of Urology (EAU) Guidelines on the Treatment of Prostate Cancer. Although more effective AR antagonists have evolved, tumor cell resistance has also evolved, culminating in the almost inevitable progression to fatal CRPC. Recent reports have suggested that ADT may lead to resistance to PCa denervation (4). Therefore, alternative methods are required for ADT to prevent the development of CRPC.

It is known that estrogen receptors (ERs) and progesterone receptors (PRs) play important roles in breast cancer (5). Currently, ERs and PRs targeted therapies are effectively used in breast cancer (6–8), but little information is available in PCa-related targeted therapies. The conventional thinking on synthetic estrogens (mainly hexestrol) is limited by their greater side effects, such as breast feminization, edema, and thrombosis, and also possibly by the lack of reliable and prospective clinical studies. However, numerous researches have suggested that ERs and PRs possibly become novel treatment modalities in PCa. Current results suggest that ERα is generally considered as an oncogene (9), while ERβ usually is a tumor suppressor (10). While relevant research has been completed or is in progress, ERs seem not to have been appropriately taken in clinical treatment. There are still no consistent outcomes on the mechanism of action of PRs. Most studies confirm the oncogenic function of PRs (11, 12), and only a few studies show the tumor-suppressive function (13). It is important to summarize the current research hotspots and trends, but there is no bibliometric analysis of ERs and PRs in PCa.

The study addressed the following questions about ERs and PRs in PCa: 1) What were current trends, and future directions? 2) Which were the most influential authors, sources, institutions, and countries? 3) Which were the most valuable publications? According to the results, not only could quickly find core authors, leading countries, and most influential articles, but also identify the current hotspots and future directions.

## 2 Materials and methods

### 2.1 Data analysis

First, we obtained a complete record of all the literature from the Web of Science (WOS), including titles, abstracts, countries, institutions, journals, authors, cited references, and keywords, all of which were downloaded as a txt file and imported into VOSviewer software, Citespace software, and Bibliometrix. The “Citation Report” function was used to obtain all cited articles, the number of self-cited articles, and citations per item.

Bibliometrix was utilized for scientific and visual analysis of data from WOS data. Key information was obtained for all publications, including the number of countries, institutions, sources, authors, documents, and references. We performed a preliminary descriptive analysis of the results, including country collaboration, authors (author production over time, and most relevant authors), sources (source dynamics, and most relevant sources), and most cited documents. VOSviewer is a visualization software for document knowledge units based on visualization of similarities technology, with the unique advantages of easy mapping and beautiful images (14). VOSviewer was used to analyze the most prolific countries, organizations, and sources. As well as the visual analysis of countries, citations and keywords were also generated from VOSviewer. Citespace (version 6.1.R2) is a visualization analysis software that analyzes trends and dynamic changes in the scientific research literature, and can quickly pinpoint key points in the field (15). Papers showing the keywords and references with strong citation bursts were derived from Citespace.

### 2.2 Data sources

On May 1, 2022, we searched for articles related to ER, PR, and PCa from the WOS core database of Clarivate Analytics, excluding the Arts and Humanities Citation Index (2003-present) version. Medical Subject Headings (Mesh) and the terms “estrogen receptor,” “progesterone receptor,” and “prostate cancer” were utilized as retrieval tactics. The formula for retrieval included the following: TS= (“prostate neoplasm” OR “prostatic neoplasm” OR “prostate cancer” OR “prostatic cancer”) AND TS= (“estrogen receptor” OR “estrogen nuclear receptor” OR “progesterone receptor” OR “progestin receptor”).

**Figure 1:**
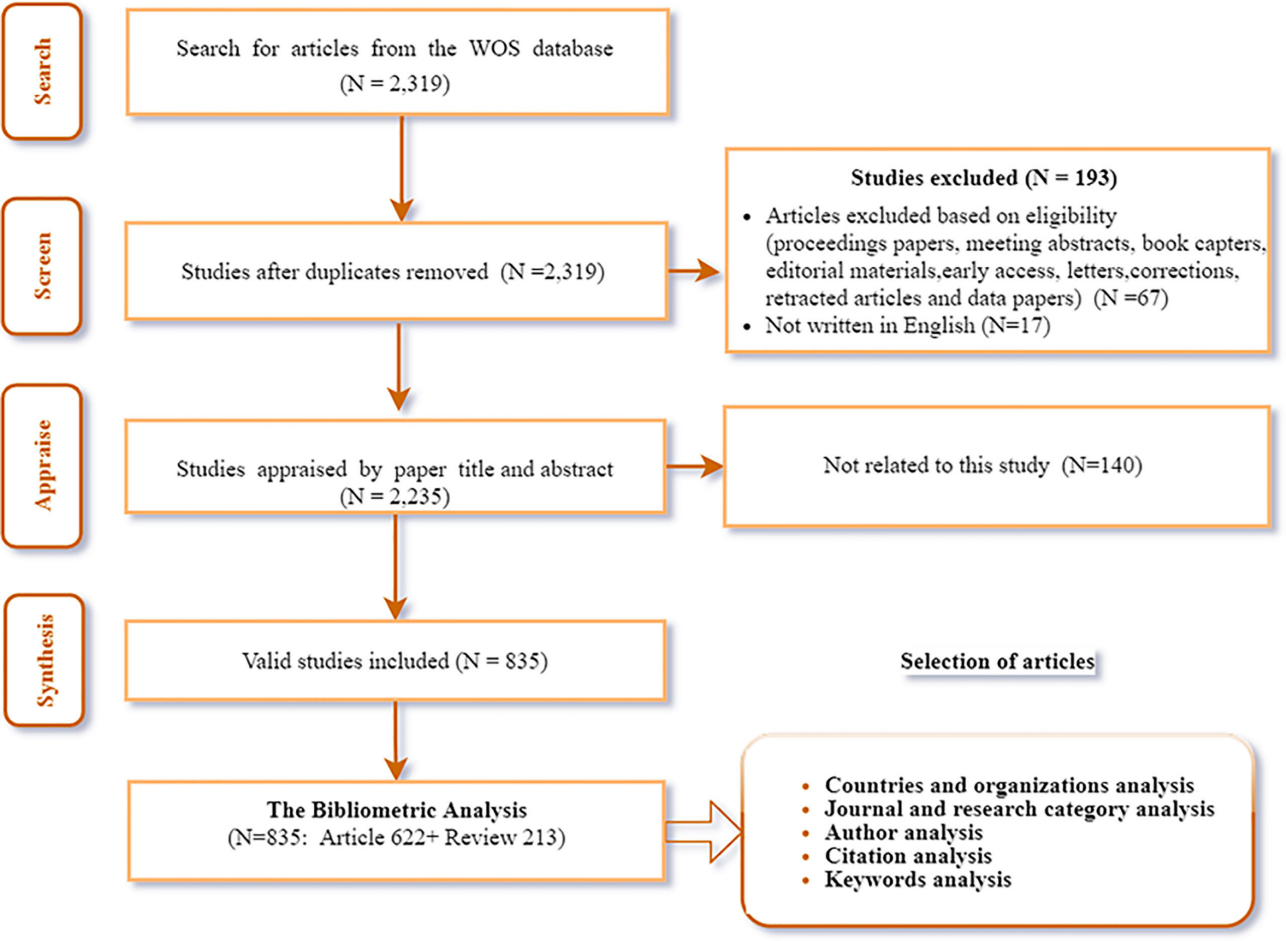
The articles selection process for bibliometric analysis.

There were 2,319 documents generated in this field from January 1, 2003, to May 1, 2022. By excluding non-article or non-review article types and restricting the language to English, 2235 articles were retained. Following that, a manual review was conducted to examine the contents of each article to eliminate irrelevant publications that were not relevant to this study. As follows were the selection criteria: (1) The research topic was unrelated to PCa; (2) The research direction was not ER or PR. Finally, 835 useful articles were retained for the bibliometric analysis (**Figure 1**).

## 3 Results

### 3.1 Publication trends

**Figure 2:**
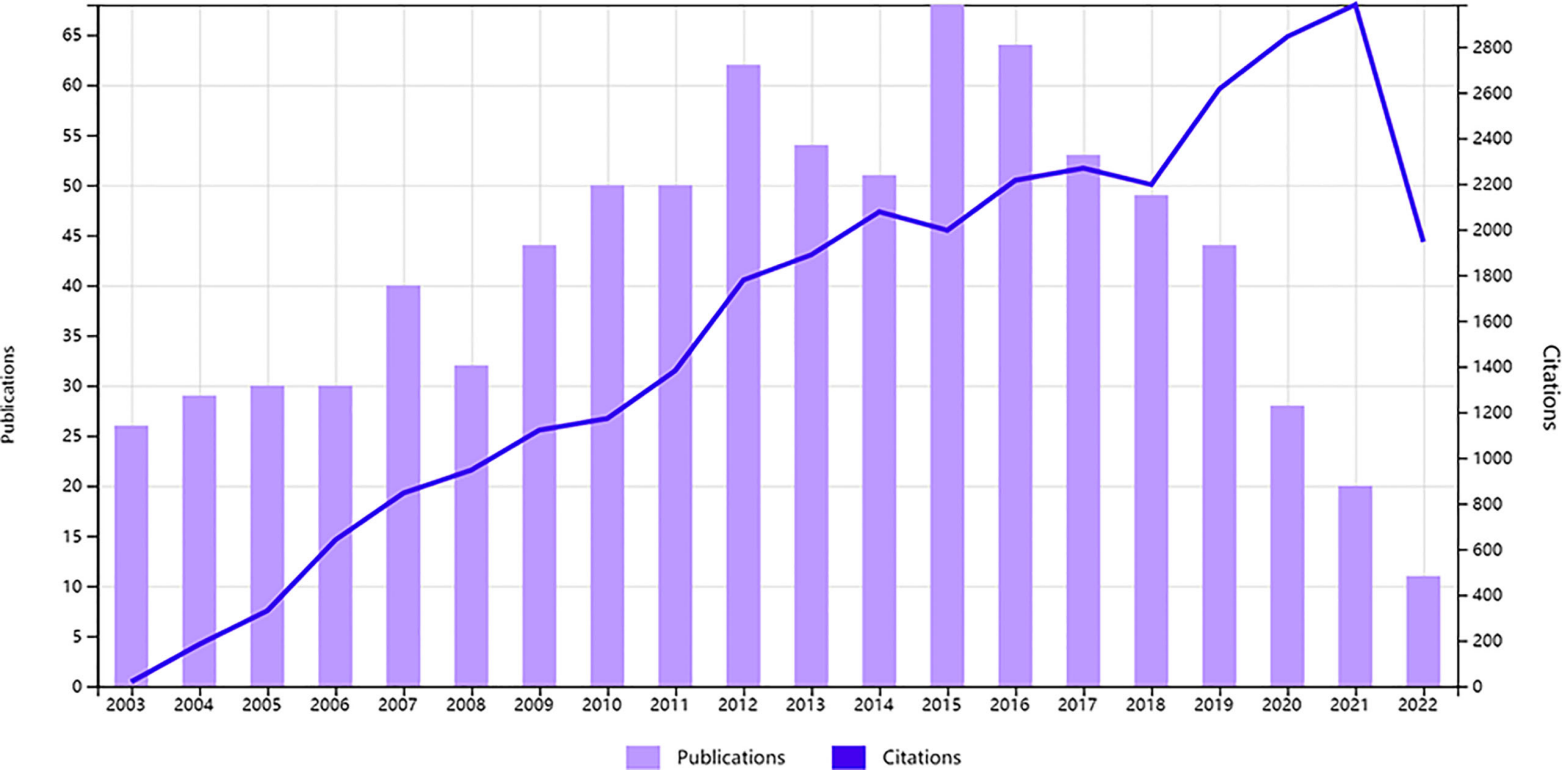
Annual production and times cited from 2003 to 2022.

With the help of Bibliometrix, the 835 papers used in this study were from 4406 authors from 1050 organizations in 51 countries, and published in 323 journals up to May 2022. The published articles were broadly divided into two periods: 2003-2015 and 2016-2021 (**Figure 2**). In the first period, the number of publications increased yearly and exceeded 50 publications in 2010, indicating that this research area has been receiving more attention. The peak number of relevant publications was recorded in 2015 (n = 68). However, the number of published papers gradually decreased in the second period, possibly indicating that this research field has reached a bottleneck. Prospective research, scientific analysis, and forward direction are urgently needed.

### 3.2 Analysis of countries and organizations

In the last 20 years, we retrieved 835 papers from WOS with 50 countries contributing to the study field from both developing and developed countries. **Table 1** lists the top 10 countries. The United States (USA) had the most publications (n = 394, 47.19% of total), followed by China (n = 110, 13.17%), Japan (n = 60, 7.19%), Italy (n = 56, 6.71%), and Germany (n = 48, 5.75%). The American publications had the most citations (n = 18,923), followed by Sweden (n = 2,926), China (n = 2,718), Japan (n = 2,579), and Italy (n = 2,546). Average article citations over 30 were all from developed countries.

**Table 1:** Top 10 cited countries contributing in the field.

Country	Documents	Citations	Average article citations
USA	394	18,923	48
China	110	2,718	25
Japan	60	2,579	43
Italy	56	2,546	45
Germany	48	2,299	48
Canada	46	2,098	46
Sweden	43	2,926	68
England	33	1,686	51
France	33	1,302	39
India	26	676	26

In the co-authorship of countries analysis by VOSviewer (**Supplementary Figure S1**), the number of countries with over 5 articles was 32. The top 5 countries, ranked by total link strength (TLS), were the USA (TLS = 184), Germany (TLS = 58), Canada (TLS = 57), China (TLS =57), and Sweden (TLS = 54). Total link strength is a way to measure the strength of relationships between nodes. The calculated numbers could reflect strong connections between authors, countries, or other identified elements (16). In other words, the more nodes, the more contributions in the field.

There were 1,050 institutions in this field. Analyzing organizations with over 5 articles, we obtained results for 65 organizations, with Karolinska Institutet contributing the most significant number of publications (n = 26), followed by Baylor College of Medicine (n = 23), University of Cincinnati (n = 19), University of Illinois (n = 18) and University of Houston (n = 18) (**Table 2**). The top 10 organizations of document volume were all located in the USA, Canada, and Sweden.

**Table 2:** Top 10 organizations contributing to this area of research.

Organization	Documents	Citations	Total link strength	Country
Baylor College of Medicine	23	1489	8	USA
Karolinska Institutet	26	2227	27	Sweden
University of Illinois	18	1203	13	USA
Harvard University	15	981	17	USA
University Of Cincinnati	19	765	22	USA
University of San Francisco	14	994	13	USA
University of Rochester	14	621	11	USA
The University of British Columbia	12	549	14	Canada
University of Houston	18	1365	22	USA
University Of Washington	16	461	27	USA

### 3.3 Analysis of journal and research category

All papers were published in 323 journals. The top 10 journals with the highest publications in this field published 222 documents, representing 26.59% of all documents (**Table 3**). Prostate published the most significant number of documents (n = 36). The second-ranked source was PLoS One (n = 30). Journal of Steroid Biochemistry and Molecular Biology ranked third (n = 26), followed by Molecular and Cellular Endocrinology (n = 24). Prostate was the most cited (1,250 citations), followed by Cancer Research (1,177 citations), and had the highest impact factor (IF = 13.31).

**Table 3:** The most influential source in this field.

Source	Documents	Citations	IF (2022)	JCR (2022)
Prostate	36	1250	4.01	Q2
PloS One	30	788	3.75	Q2
Journal of Steroid Biochemistry and Molecular Biology	26	620	5.01	Q2
Molecular and Cellular Endocrinology	24	630	4.37	Q2
Molecular Endocrinology	21	815	4.87	Q2
Endocrine-Related Cancer	20	926	5.90	Q2
Cancer Research	17	1177	13.31	Q1
Journal of Biological Chemistry	17	759	5.49	Q2
Oncotarget	16	414	/	/
Endocrinology	15	438	5.05	Q2

In 2018, Oncotarget was removed from the SCI (Science Citation Index). Therefore, the symbol "/" indicates that it is not included in the SCI.

Co-citation analysis of sources was performed in VOSviewer. There were 38 sources with citations over 300, and the max lines were set to 600 to obtain Network Visualization (**Supplementary Figure S2A**). The element’s size depended on the number of citations (the more citations, the bigger the node), and the different colors represented the different clusters. Density visualization showed that density depended on the number of elements in the surrounding area and the importance of these elements: The higher the density was, the closer it was to yellow (**Supplementary Figure S2B**). The general clustering trend of sources in the two clusters was that the red group represented the sources in the cancer endocrine field, and the green group represented the sources in the molecular and cellular fields. All clusters almost covered the current topics in our research domains.

The H-index is usually applied to depict the journal’s impact (17). Prostate had the maximum H-index of 21, followed by PLoS One (H-Index = 20), Molecular and Cellular Endocrinology (H-index = 18), Cancer Research (H-Index = 17), and Journal of Biological Chemistry (H-Index = 15) (**Supplementary Table S1**). Bradford’s law was used to test the quantitative distribution pattern of articles in journals, contributing to searching for core journals (18). According to the source clustering through the Bradford’s Law function of Bibliometrix (**Supplementary Table S1**), the above journals (Prostate, PLoS One, Molecular Endocrinology, Cancer Research, and Journal of Biological Chemistry) were all in zone 1, which further validated the significance of these journals.

**Figure 3:**
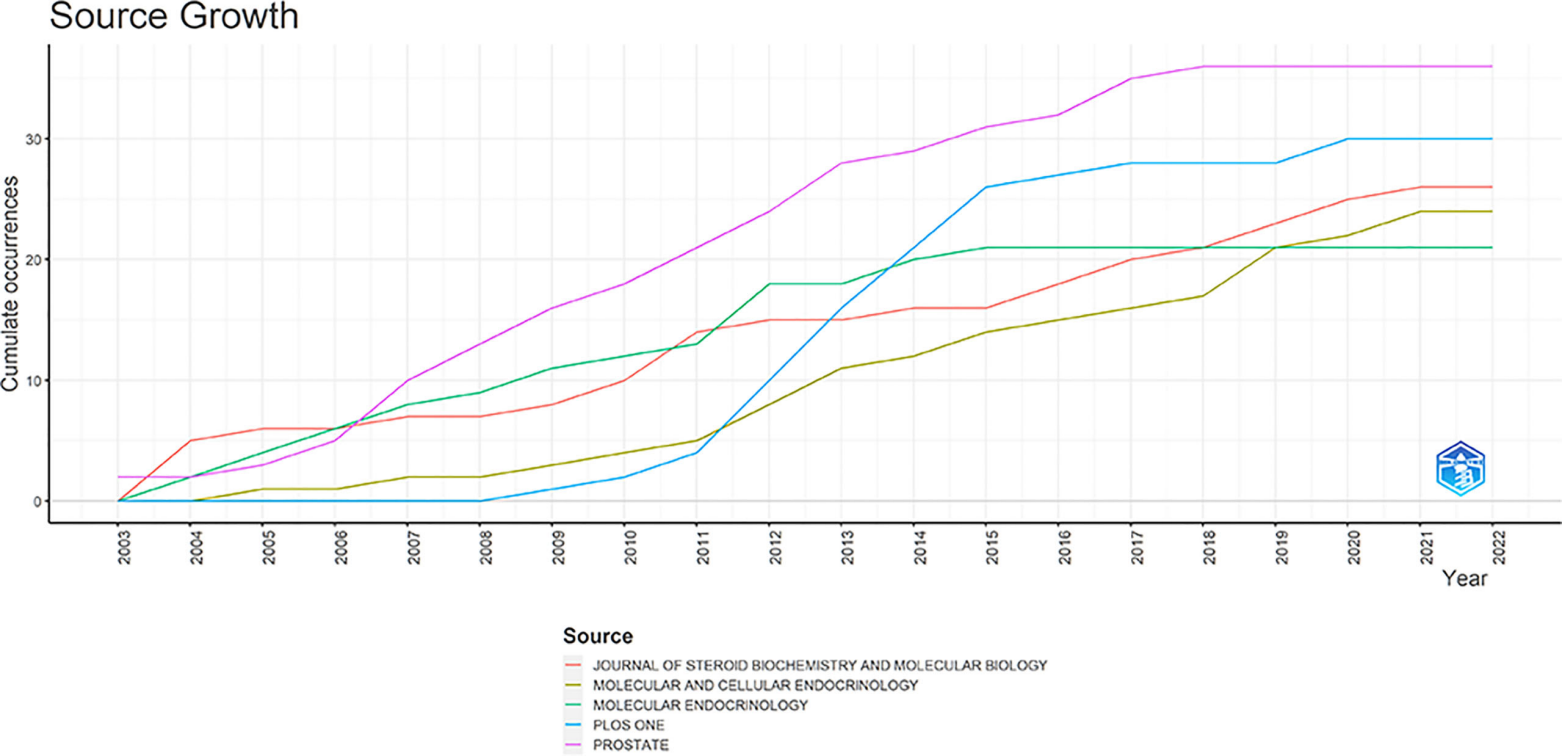
Source dynamics over the years associated with the scope of this study.

According to the source dynamics function of Bibliometrix (**Figure 3**), it could be concluded that the field of ERs and PRs related to PCa was on the rise with the year. However, in recent years it has been close to stagnation, indicating an urgent need for novel topics and directions to promote the development in this field. Two journals were worth noting. One was Prostate, has been ranked first in 2006. The other one was PLoS One, which has increased its publication volume year by year since the first article in the field was reported in 2008, having surpassed the second-ranked Molecular Endocrinology in terms of number of articles published in 2013. PLoS One was expected to surpass Prostate to become the first in the field.

A total of 61 research categories were covered by the Analysis Results function of WOS. The top 5 subject categories in terms of number of publications were Oncology (n = 241), Endocrinology Metabolism (n = 194), Biochemistry and Molecular Biology (n = 181), Cell Biology (n = 140), and Urology Nephrology (n =74) (**Supplementary Figure S3**). Regarding publishers, the most frequent publishers were Elsevier (n = 197 publications), Springer Nature (n = 99), Wiley (n = 99), American Association for Cancer Research (n = 36), and Oxford University Press (n = 33).

### 3.4 Analysis of authors

Across all datasets, 4,406 authors contributed to the 835 publications, where authors appeared a total of 5477 times, and each document had an average number of authors of 6.56. There were 18 authors of single-authored documents. Among the highly productive authors (**Table 4**), the top 5 were Jan-Ake Gustafsson (n = 21 articles, 1828 citations), Shuk-Mei Ho (n = 18, 1043 citations), YuetKin Leung (n = 11, 508 citations), Warner, Margaret (n = 10, 690 citations), and Satoshi Inoue (n = 10, 333 citations). Among the most productive authors, four authors had an H-index over 10, namely Jan-Ake Gustafsson and Shuk-Mei Ho were both top 1st place (H-Index = 16), followed by YuetKin Leung and Satoshi Inoue (H-Index = 10). Considering the difference in seniority of researchers, the M-index is a modification of the H-index of time, helping to recognize successful researchers (17). The top two M-Index rankings were Jan-Ake Gustafsson and Shuk-Mei Ho.

**Table 4:** The most influential authors in this field.

Element	NP	TC	TC/NP	H-index	G-index	M-index	PY start
GUSTAFSSON JA	21	1828	87.05	16	21	0.842	2004
HO SM	18	1043	57.94	16	18	0.842	2004
INOUE S	10	333	33.30	10	10	0.5	2003
LEUNG YK	11	508	46.18	10	11	0.526	2004
CASTORIA G	9	363	40.33	9	9	0.563	2007
RICKE WA	9	703	78.11	8	9	0.4	2003
WARNER M	10	690	69.00	8	10	0.421	2004
ZHANG Y	8	346	43.25	8	8	0.5	2007
FUJIMURA T	7	209	29.86	7	7	0.35	2003
MIGLIACCIO A	7	261	37.29	7	7	0.438	2007

Number of Publications (NP), Number of Publications (TC), Publication year (PY)

**Figure 4:**
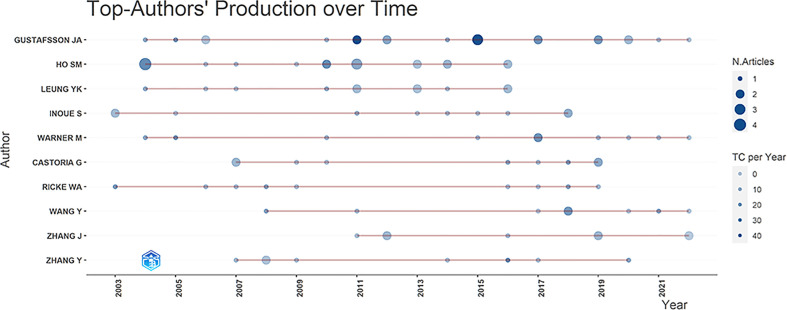
Authors’ production over time in this area.

The top-the Authors’ Production Over Time (**Figure 4**) presented the top authors’ publications in the last 20 years. The graph point size was proportional to the overall annual quantity of citations. Since 2018, there has been a general decline in article output, except for Jan-Ake Gustafsson, Margaret Warner, Wang Yue, and Ju Zhang, who have maintained a relatively stable output. Shuk-Mei Ho, and YuetKin Leung published increasingly in the first 10 years but decreasingly in the last 5 years. The outputs of Satoshi Inoue, Gabriella Castoria, William A. Ricke, and Yin Zhang were inconsistent in the previous 10 years.

The three-field plot showed the relationship between the top countries (middle), affiliations (left), and the most productive authors (right) (**Supplementary Figure S4**). The strength of the connection was represented by the number of the lines. The USA was the most connected country (n = 36), followed by China (n = 22), and Canada (n = 15). The most productive organizations from the USA were the University of California and the University of Pittsburgh (both are 11/36 = 30.56%), followed by Harvard University and the University of Massachusetts (both are 10/36 = 27.78%). The top 2 authors with the strongest trend of collaboration between countries were Brow (n=8) and Risbridger (n = 6).

### 3.5 Analysis of citations

The number of citations reflects the influence of articles in the field (19). The total citing documents were 23,929 of all retrieved articles, including 23,394 without self-citations (97.76% of all citing documents) and 5,35 self-citations. The number of cited articles was 31,451, which included 28,908 without self-citations. The average number of times cited per document was 37.67. Highly cited papers provided us with frontier research areas. **Table 5** provides the top 10 articles based on total citations of ERs, PRs and PCa studies. In 2006, the most cited paper was “*Estrogen receptors and human disease”* by Deroo BJ, published in the Journal of Clinical Investigation. Among the top 10 articles, 8/10 of the top 10 articles were published in the previous 10 years and still cited today, which meant they had a high reference value. Based on average citations per year, *“Sustained proliferation in cancer: Mechanisms and novel therapeutic targets”* by Feitelson MA in 2015 (39.5 citations per year) and *“Estrogen receptors alpha (ER alpha) and beta (ER beta): Subtype-selective ligands and clinical targets”* by Paterni I in 2014 (32.78 citations per year). These two articles have summarized the relatively new field, focusing on clinical potential and novel therapeutic targets.

**Table 5:** Top 10 publications based on citations of ERs, PRs, and PCa research.

Title	Source	Year	DOI	Citations	Average per Year
Estrogen receptors and human disease	JOURNAL OF CLINICAL INVESTIGATION	2006	10.1172/JCI27987	898	52.82
The different roles of ER subtypes in cancer biology and therapy	NATURE REVIEWS CANCER	2011	10.1038/nrc3093	460	38.33
Cytochrome P450-mediated metabolism of estrogens and its regulation in human	CANCER LETTERS	2005	10.1016/j.canlet.2004.10.007	394	21.89
Normal and cancer-related functions of the p160 steroid receptor co-activator (SRC) family	NATURE REVIEWS CANCER	2009	10.1038/nrc2695	351	25.07
Loss of ER beta expression as a common step in estrogen-dependent tumor progression	ENDOCRINE-RELATED CANCER	2004	10.1677/erc.1.00800	344	18.11
Interaction of nuclear receptors with the Wnt/beta-catenin/Tcf signaling axis: Wnt you like to know?	ENDOCRINE REVIEWS	2005	10.1210/er.2003-0034	324	18
Sustained proliferation in cancer: Mechanisms and novel therapeutic targets	SEMINARS IN CANCER BIOLOGY	2015	10.1016/j.semcancer.2015.02.006	316	39.5
Estrogen receptors alpha (ER alpha) and beta (ER beta): Subtype-selective ligands and clinical potential	STEROIDS	2014	10.1016/j.steroids.2014.06.012	295	32.78
ER beta Impedes Prostate Cancer EMT by Destabilizing HIF-1 alpha and Inhibiting VEGF-Mediated Snail Nuclear Localization: Implications for Gleason Grading	CANCER CELL	2010	10.1016/j.ccr.2010.02.030	294	22.62
Role of the stromal microenvironment in carcinogenesis of the prostate	INTERNATIONAL JOURNAL OF CANCER	2003	10.1002/ijc.11335	292	14.6

The top 25 references with the strongest citation bursts were obtained from Citespace (**Supplementary Figure S5**). Each red or short blue line stood for a year, and the red represented the burst year. In the last 20 years, 36% of references (9/25, 36%) had a citation burst from 2008 to 2012, followed by 2003 to 2007 (8/25, 32%), 2013 to 2017 (7/25, 28%), and 2018 to 2022 (1/25, 4%). The strongest reference to obtain a burst (Strength = 9.82) was a paper by Mak, P et al., with a citation burst occurring between 2011 and 2015 (20). In addition, the review written by Helmut Bonkhoff in 2018 was continuously cited until 2022 (21).

### 3.6 Analysis of keywords

**Figure 5:**
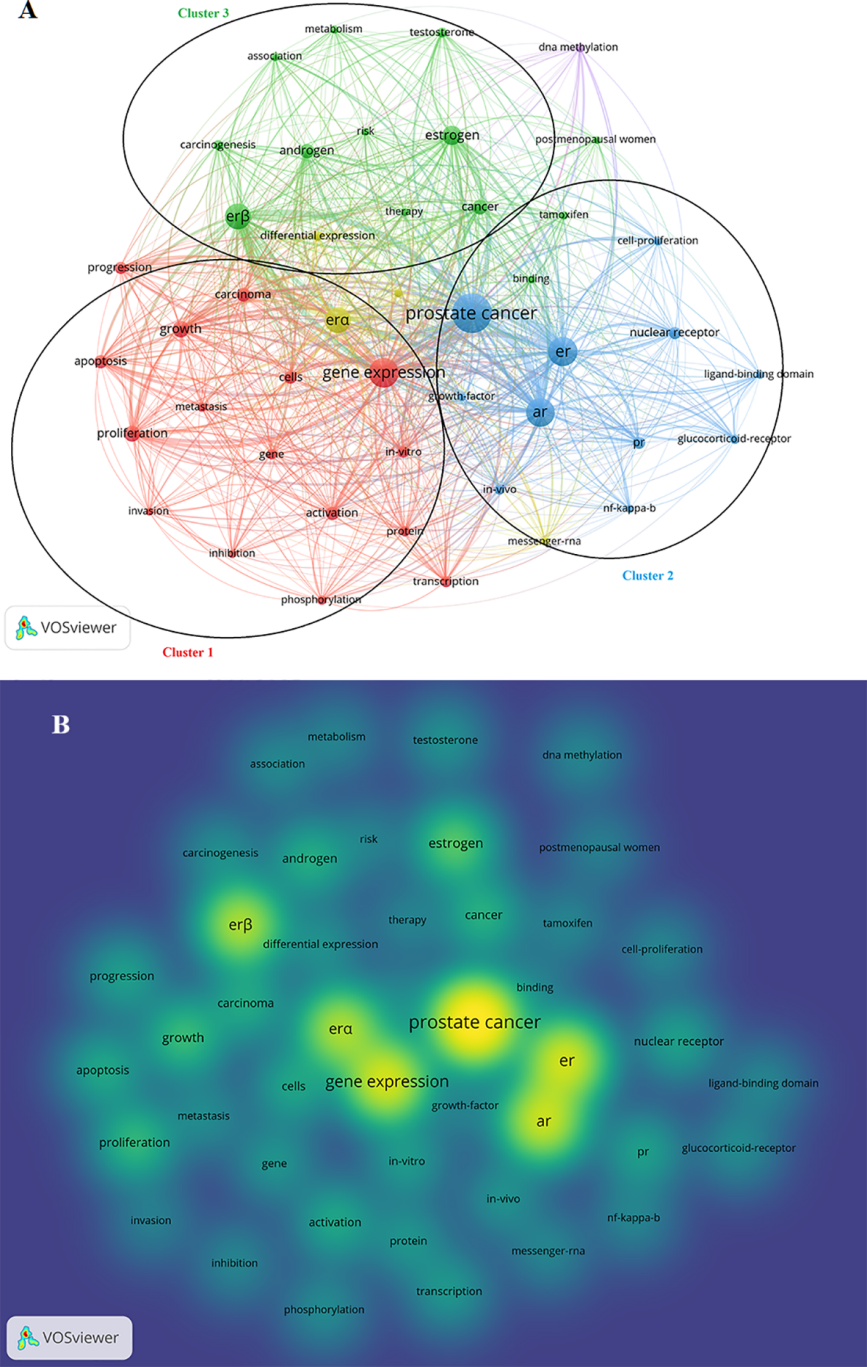
Co-citation analysis of references in the field. **(A)** Network map of co-citations among references cited over 20 times. **(B)** Density map of co-citations among references cited over 20 times.

The co-occurrence analysis of all keywords in VOSviewer had a total of 3,970 keywords and 67 keywords with over 20 occurrences. By manually merging synonyms and removing meaningless keywords (such as breast-cancer and breast etc.), 45 popular keywords were obtained. The most frequently used keywords were prostate cancer (n = 499), gene-expression (n = 291), AR (n = 263), and ER (n = 341), where ERβ (n = 219) and ERα (n = 215) also further determined the importance of ER (**Supplementary Table S2**). The network visualization indicated that 45 popular keywords were sorted into 5 clusters (**Figure 5**), which assisted in determining the orientation of the field. Cluster 1, with 16 red items, illustrated the primary microscopic mechanisms (genes, gene expression, phosphorylation, transcription, apoptosis, etc.) and macroscopic manifestations of tumors (growth, metastasis, invasion, progression, proliferation, inhibition, etc.). The green cluster 2, with 13 items, showed an association between the sex hormone (such as estrogen, androgen, and testosterone) carcinogenesis and cancer therapy. The blue cluster 3, with 11 items, described the relationship between receptors (ER, PR, glucocorticoid receptors, and nuclear receptors) and PCa. The density visualization map provided a visual representation of the research priorities in the field (**Figure 5B**). The keywords “PCa,” “gene expression,” “AR,” “ER,” and “ER-related subtypes” seemed to be hot keywords that were active among the studies in the last 20 years.

**Figure 6:**
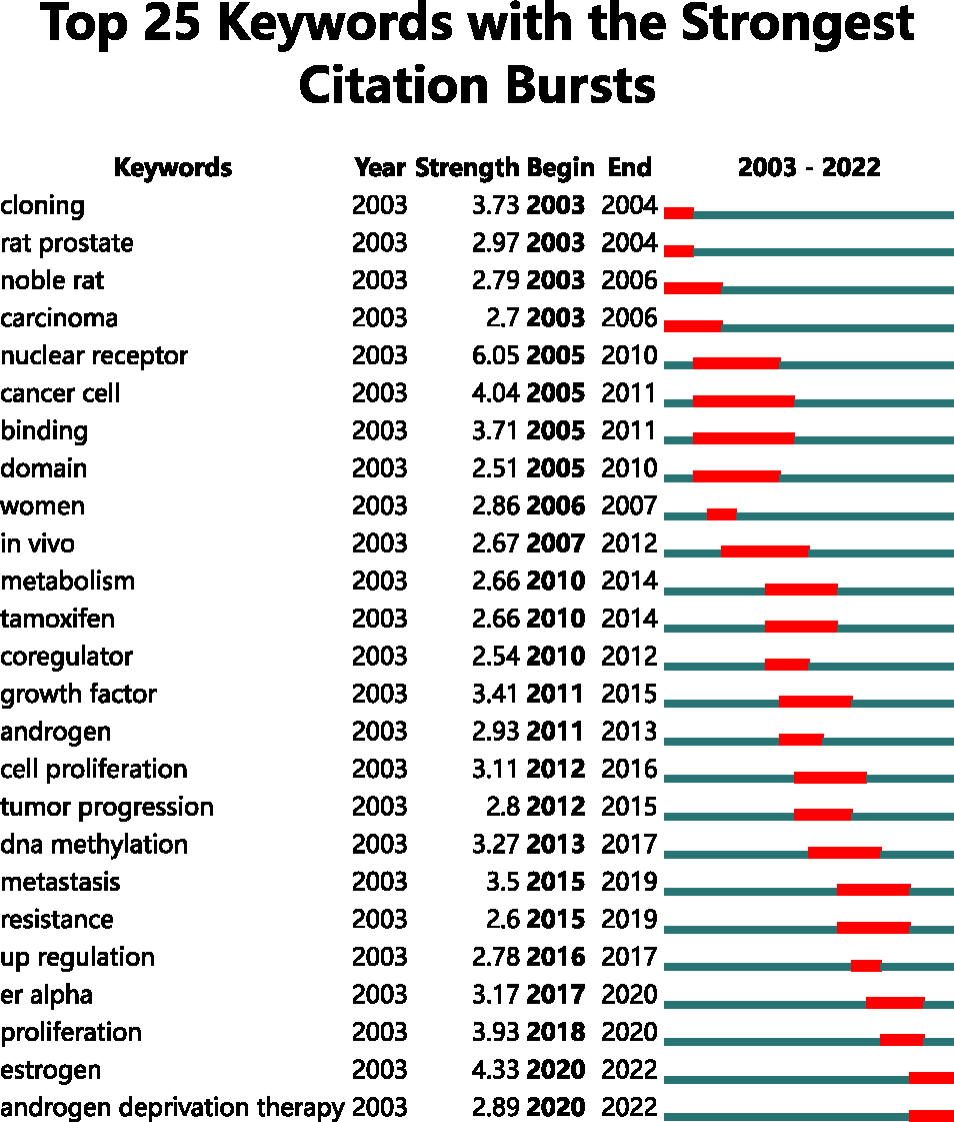
Top 25 keywords with the strongest citation bursts.

The burst keywords from Citespace provided an access to research trends in recent years and predicted possible future trends (**Figure 6**). The middle seventeen keywords emphasized the trends of numerous studies from 2005 to 2016, which mainly focused on molecular mechanisms in the middle, such as DNA methylation, up-regulation, and growth factor. The first four keywords showed that the field might be in the animal testing stage. The four recent keywords indicated a lot of attention, meaning they were the focus of current research. The keywords (“estrogen” and “androgen deprivation therapy”) continued to be effective until 2022. The keyword “nuclear receptor” received the strongest burst (strength = 6.05) from 2005 to 2010 citation burst.

## 4 Discussion

### 4.1 Global research trends in estrogen receptor and progesterone receptor on prostate cancer

The study conducted a bibliometric analysis of ERs, PRs and PCa with the help of Bibliometrix, VOSviewer, and Citespace. The search strategy and filtering resulted in 835 papers, including 4406 authors, 1050 organizations, 51 countries, 323 journals, and 4,406 authors.

Our research on the relationship between ERs, PRs and PCa started in 2003 with an upward and then downward trend in the number of publications. The peak number of publications in 2015 (n = 67) was 3.4 times higher than the number of publications in 2020 (n = 20). Half of the top 10 highly cited publications were published in the beginning phase (2003-2006), which was probably a time of rapid growth in this research field. All the above findings demonstrated that ER, PR and PCa used to attract a lot of attention from researchers, but in recent years the paper output has declined significantly. It is possible that the research field has hit a bottleneck. For example, whether PCa cell line models express ERβ and the use of different concentrations of estrogen will likely lead to dual activation of ER or regulation of other pathways. These issues remain contentious because of the absence of specific ERβ antibodies (22). It urgently needs forward-looking research, scientific analysis, and providing a way forward.

In total, we introduced 50 countries that have contributed to the development of this field, of which the USA was the pioneer, with the most articles (n = 394) and the most citations (n = 18,923). Eight of the 10 most productive institutions were located in the USA. The countries with average article citations more than 30, and the top 10 publishers were all from developed countries. These results may be attributed to the widespread application of prostate-specific antigen (PSA) testing at the end of the 1980s (23), which led to increased incidence and extensive input of scientific capital. Several developing countries, like India and China, have also made partial achievements in this field. In particular, China has performed well with the second-highest number of publications (n = 110) and the third-highest number of citations (n = 2,926). However, China was relatively weak regarding average citations per item, indicating a lack of high-quality research results. It may be due to China’s relatively late start in this field and lack of experience in international cooperation. Based on GLOBOCAN 2020 data, PCa incidence and death in China account for 8.2% and 13.6% worldwide, respectively. The country shoulders a huge burden of cancer incidence and mortality. Thus, it is proposed that China and its institutions should strengthen cooperation and communication with European and American research institutions to promote progress in this field.

The journals’ analysis showed that researchers focused on the cancer endocrine and molecular fields. Based on the number of articles, the top 5 journals were shown in **Table 3**: Prostate (n = 36), Journal of Steroid Biochemistry and Molecular Biology (26 articles), Molecular and Cellular Endocrinology (n = 24), Molecular Endocrinology (n = 21), and Endocrine-Related Cancer (n = 20). PLoS One (n = 30) has become the second choice in the field in 2013, partly because it is an open-access journal, which shows that the development of open-access journals in recent years has immensely promoted the advancement of research in this field. Although researchers are still split on the best path to open-access, the concept that research results should be freely shared is broadly accepted (24). The top 5 research categories were Oncology, Endocrinology Metabolism, Biochemistry and Molecular Biology, Cell Biology, and Urology Nephrology. This finding demonstrated the importance of the cancer endocrine and molecular fields again.

The H-index showed that Jan-Ake Gustafsson, Shuk-Mei Ho, Satoshi Inoue, YuetKin Leung, and Gabriella Castoria were the five most accomplished authors in this field. Jan-Ake Gustafsson had the greatest H-index, M-index, publications, and citations, specializing in the role of nuclear receptors in the growth, development, and disease states of various tissues. Jan-Ake Gustafsson booked *“The differential role of ER subtypes in cancer biology and therapy”* in 2011, the second most cited publication (460 citations), and was widely recognized (25). The author argues that ERβ (especially ERβ1) exerts cancer-suppressive effects, such as oppressing the proliferative effects of ERα, inhibiting epidermal growth factor receptor (EGFR) and suppressing tumor bone metastasis (26–29). Therefore, ERβ1 selective agonist is considered a possible strategy for treating PCa (30).

Jan-Ake Gustafsson remained active in this field and continued contributing to the development of the field (**Figure 4**). The second H-index was Shuk-Mei Ho, whose research focused on estrogen signaling in PCa. She published her first article on estrogens and PCa in 2004 (165 citations) (31). Subsequently, her research demonstrates the metastasis-promoting role of ERβ2 and ERβ5 in PCa in 2011 (32), and proves that the first discovery of a new non-genomic tumor suppressor “has-miR-765”, which may serve as a novel treatment option for PCa (33).

### 4.2 Literature review, hotspots, and frontiers

The analysis of citations and keywords enables us to quickly pinpoint the research themes and the core content of publications. It also contributes to our awareness of the field’s current research hotspots and frontiers.

The 10 most cited papers summarized the structure of the existing knowledge framework concerning ER-related subtypes and PCa (25, 34–36). The most cited publication, namely “Estrogen receptors and human disease,” was published by Deroo BJ et al. in the Journal of Clinical Investigation in 2006 (IF = 19.456) and was cited 898 times (34). This article concluded that ERα was oncogenic function only in the prostate stroma; however, ERβ presented in both the luminal epithelial and stroma, and has a cancer-suppressive function. This idea is widely accepted and lays the foundation for subsequent ERs-related studies. Four of the top 10 publications described several signaling pathways and key protein interactions in ER, such as cytochrome P450 (CYP), the steroid receptor co-activator (SRC), and Wnt/β-catenin (20, 37–39). Among them, “Cytochrome P450-mediated metabolism of estrogens and its regulation in human” by Mak P et al. received the strongest citation burst (intensity = 9.82). In an average of more than 30 citations per year, three relatively new articles in this field gradually focused on the shift from mechanisms to clinical treatments (25, 36, 40). However, only minimal work has been done in clinical applications, so further research is needed.

According to the keyword density map (**Figure 5B**), The keywords “PCa,” “gene expression,” “AR,” “ER,” and “ER-related subtypes” seemed to be hot keywords that were active among the studies in the last 20 years. The keyword cloning hints at the fact that ERα was cloned in the 80s, and ERβ was discovered by the Gustafsson group in 1996 (41). The top 10 keywords of citations were overwhelmingly dominated by ER and its related subtypes. It is reported that there are five subtypes of ERβ. ERβ1 is wild-type, and the others are splice variants designated as ERβ2-5 (42). ERβ1 is the unique full-function receptor, while ERβ2, ERβ4, and ERβ5 counteract ERβ1 (43), with the largest degree of dominant-negative regulation occurring between ERβ1 and ERβ2 (44). Namely, ERβ1 has the predominant tumor-suppressive effect. On the contrary, ERβ2, ERβ4 and ERβ5 splice variants have oncogenic functions (45). Among them, ERβ2 not only has oncogenic function, but also participates in bone metastasis of tumor, which plays an opposite role to the tumor-suppressive function of ERβ1 (46). ERβ is considered as a tumor-suppressive receptor, and favoring its activation would lead to PCa regression. Conversely, ERα stimulates cell proliferation of PCa and its expression is higher as the tumor progresses (9). Thus, ERα antagonists or ERβ agonists emerge as an attractive alternative therapy for CRPC. It can be found that most of the burst words concentrated in 2010-2017 (**Figure 6**), where mechanisms and signaling pathways (such as coregulator, growth factor, DNA methylation, and up regulation) occupy the leading position and also enter the peak period of publication at the same time. Since 2020, the keywords “estrogen” and “androgen deprivation therapy” were the citation burst. Therefore, the study focus has switched to medical treatment. It is known that ADT is the predominant treatment for PCa, and ADT is initially effective but causes eventual CRPC. An explanation could be that AR is expressed only in epithelial cells of the prostate lumen but not in basal cells. This localization pattern is well explained by the fact that ADT can inhibit AR-positive PCa proliferation but has no effect on basal cell proliferation/differentiation. In addition, dihydrotestosterone (DHT) can be endogenously metabolized to 5α-androstane-3β. ADT blocks DHT production and then decreases endogenous estradiol (5α-androstane-3β), a ligand for both ERs (47). Endogenous estradiol binds to both ERβ and ERα, having a slightly higher affinity for ERβ (48). Consequently, ADT can inhibit AR signaling, and predominantly inhibit the tumor-suppressive function of ERβ. A recent clinical study by Jan-Ake Gustafsson showed that ADT, such as finasteride (5α-reductase inhibitor), enzalutamide (AR blocker), and abiraterone (androgen synthesis inhibitor), promoted EGFR nuclear translocation and increased PCa proliferation on the treatment of PCa. Another result from this study showed that treatment with isoflavones (ERβ agonists) inhibited finasteride-induced EGFR nuclear translocation during prostatic hyperplasia (BPH) treatment (28). Therefore, ERβ receptor agonists with ADT may be used as a new modality to treat PCa since nuclear EGFR predisposes to CRPC. Biochemical recurrence rates in bone metastatic PCa have been reported to be improved by the combination of toremifene (a selective estrogen receptor modulator) and ADT (49), and fulvestrant (a selective estrogen receptor down regulator) is able to reduce PSA levels in some patients without any detectable toxicity (50). It is exciting that the combination of estrogen with ADT will potentially serve as a new treatment strategy for PCa. However, it should be considered that there seems to be possible crosstalk between ERs and the G-coupled protein estrogen receptor (GPER) before estrogen is put into therapy (51). GPER is a membrane estrogen receptor, which is expressed in both PCa and normal glands (52) GPER-mediated responses have a key role in PCa, and ligand binding to GPER activates EGFR involving in the stimulation of tumor migration and invasion (53).

However, PR-related topics did not appear in the top 10 citation papers, and the keyword “PR” only appeared 49 times, suggesting that the field may not have received enough attention, or the mechanism is too complex to be known. In all stages of PCa, the presence of PRs is always linked to the high expression of ERα (54), especially in CRPC, where different levels of PRB transcripts are obtained (55). Most reports show that PRA is only present in the stroma, and PRB is expressed in both the stroma and epithelium. High PRB expression in tumor tissue is correlated with an unfavorable prognosis (11). However, Yu et al. report conflicting results, showing PRs could inhibit tumor proliferation (56), as well as that PRB is not expressed in epithelial cells (13). The difference is possibly due to the heterogeneous structure of the tumor (57). So far, Isikbay et al. have demonstrated the inhibitory effect of mifepristone on CRPC (58). However, this expected effect is not observed in small phase II clinical trials (59). PRs expression may not be directly responsible for tumor proliferation, but rather is the result of other underlying processes. In contrast to ERs, PRs-related studies are likely to be only the tip of the iceberg of complex steroid hormone interactions in PCa development, and the role of PRs in the epithelium and stroma of PCa and the potential individual roles of PRs isoforms remain to be determined.

## 5 Strengths and limitations

Several advantages are associated with our study. This study first used bibliometric analysis to assess the hotspots and frontiers in ERs, PRs and PCa research. A total of 835 publications on ERs, PRs and PCa research were obtained from WOS. Our study analyzed annual publications, countries, institutions, journals, authors, citations, and keywords. We also merged synonyms and removed irrelevant words for some keywords so that research hotspots and frontiers would be more prominent.

The limitations of this study come mainly from the following aspects. The first is the source data’s limitation which is the inability to directly merge the results from multiple databases, which may omit some important studies. The databases focus more on English language journals, resulting in a lack representation of non-English language journals. Only partial information about the literature is available, and complete information about the articles is not available. Nevertheless, WOS is one of the industry’s best digital literature resource databases and is often used by researchers for bibliometric analysis (60). The complete information of the target article can be obtained by a manual search. The second limitation is the deficiency of research tools. For example, VOSviewer can only generate a map and cannot view the node information, which can only be solved by manually searching to obtain the relevant information. Finally, most of the top 10 articles are reviews and older, probably multidisciplinary, resulting in less specificity in the research field, which requires several researchers to spend much time on scientific analysis of the literature and a more in-depth and comprehensive understanding of the field, inevitably with some subjectivity. We are confident that this bibliometric research could offer a few inspirations and viable ideas for researchers in related fields.

## 6 Conclusion

It has been widely accepted that AR has a role in PCa. Numerous researchers have recently explored the mechanisms and therapeutic benefits of ER and PR in PCa. This bibliometric study analyzed current trends, major countries, institutions, and core journals in the field, providing research findings from landmark articles, research directions, and hotspots. Relevant research in this field was deepening, from the establishment of the relationship between ER, PR and PCa, to the refinement of microscopic molecular mechanisms, and the transition to clinical treatment. This study provided some directions for researchers. ERα antagonists, ERβ agonists, and the combination of estrogen with ADT would potentially serve as a new treatment strategy for PCa. Another interesting topic is relationships between PCa and the function and mechanism of action of PRs subtypes. The outcome will assist scholars in gaining a comprehensive understanding of the current status and trends in the field, and provide inspiration for future research.

## Data availability statement

The original contributions presented in the study are included in the article/**Supplementary Material**. Further inquiries can be directed to the corresponding author.

## Author contributions

QY and XS provided the conception and design of the study. All authors participated in the development of the scheme. GH provided guidance on the use and analysis of these analytical procedures. WL obtained the dataset from Web of Science, performed the statistical analysis, and was a major contributor to writing the manuscript. MY, YL, and JL participated in the interpretation of the study results. XS revised the article critically. All authors contributed to the article and approved the submitted version.

## Glossary

**Table:**

ADT	androgen deprivation therapy
AR	androgen receptor
BPH	prostatic hyperplasia
CRPC	castration-resistant prostate cancer
CYP	cytochrome p450
DHT	dihydrotestosterone
EAU	the European Association of Urology
EGFR	epidermal growth factor receptor
ER	estrogen receptors
H-index	Hirsch index
Mesh	Medical Subject Headings
PCa	prostate cancer
PR	progesterone receptors
PSA	Prostate-Specific Antigen
TLS	total link strength
USA	The United States
SRC	the steroid receptor co-activator
WOS	Web of Science
