# Supplementary material for: Trends in estrogen and progesterone receptors in prostate cancer: a bibliometric analysis
Source: Front Oncol. 2023 Jun 9;13:1111296. doi: 10.3389/fonc.2023.1111296 (PMC10288854; doi:10.3389/fonc.2023.1111296)
Supplement: Supplementary file 1 [file DataSheet_1.pdf]

Supplementary Material

1 Supplementary Tables:

Supplementary Table S1| The top 10 H-index journals published in the field.

Element	h_index	g_index	TC	NP	PY_start	Bradford's zone
PROSTATE	21	35	1250	36	2003	Zone 1
PLOS ONE	20	27	788	30	2009	Zone 1
MOLECULAR ENDOCRINOLOGY	18	21	815	21	2004	Zone 1
CANCER RESEARCH	17	17	1177	17	2003	Zone 1
JOURNAL OF BIOLOGICAL CHEMISTRY	15	17	759	17	2003	Zone 1
JOURNAL OF STEROID BIOCHEMISTRY AND MOLECULAR BIOLOGY	15	24	620	26	2004	Zone 1
MOLECULAR AND CELLULAR ENDOCRINOLOGY	14	24	630	24	2005	Zone 1
ENDOCRINE-RELATED CANCER	13	20	926	20	2004	Zone 1
ONCOTARGET	12	16	414	16	2011	Zone 1
PROCEEDINGS OF THE NATIONAL ACADEMY OF SCIENCES OF THE UNITED STATES OF AMERICA	12	14	744	14	2004	Zone 1

Supplementary Table S2| Top 20 Keywords contributing to the field.

Rank	Keyword	Occurrences	Total link strength
1	prostate cancer	499	1654
2	gene expression	291	1146
3	er	278	957
4	ar	263	992
5	erβ	219	907
6	erα	215	812
7	estrogen	119	454
8	growth	90	411
9	proliferation	83	379
10	cancer	71	284
11	androgen	66	270
12	carcinoma	61	268
13	apoptosis	59	271
14	activation	58	247
15	cells	55	242
16	nuclear receptor	50	193
17	pr	49	191
18	progression	49	210
19	in-vitro	44	171
20	transcription	40	155

2 Supplementary Figures:

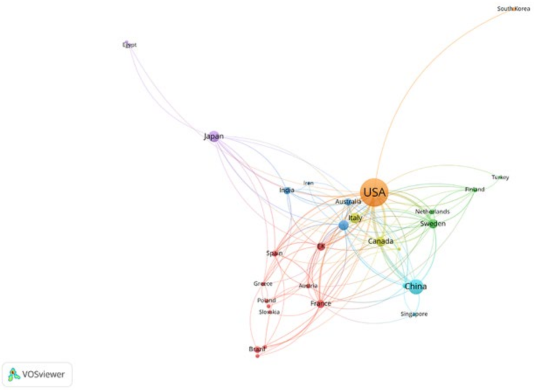

Supplementary Figure S1. Co-citation network graph of the cited references for these articles.

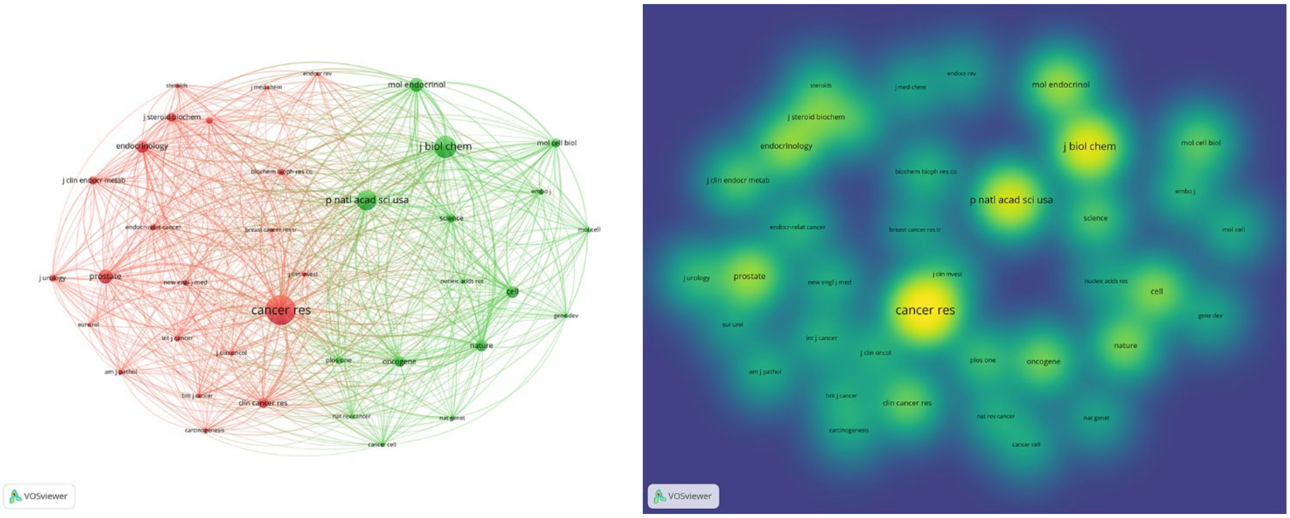

Supplementary Figure S2. Co-citation analysis of sources of these documents.

(A) Network map of co-citation between sources with more than 60 citations.

(B) Density map of co-citation between sources with more than 60 citations (yellow means more frequent)

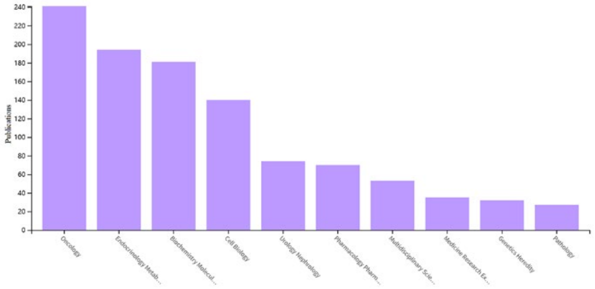

Supplementary Figure S3. Top 10 most popular research topic categories.

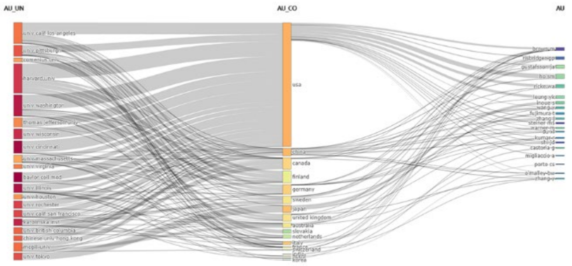

Supplementary Figure S4. Three-field plot among the most productive countries(middle) institutions(left), and top authors(right).

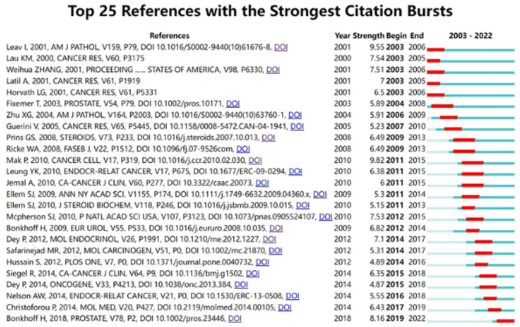
Top 25 References with the Strongest Citation Bursts

References	Year	Strength	Begin	End	2003 - 2022
Leav I, 2001, AM J PATHOL, V159, P79, DOI 10.1016/S0002-9440(10)61676-8, DOI	2001	9.55	**2003**	2006	
Lau KM, 2000, CANCER RES, V60, P3175	2000	7.54	**2003**	2005	
Weihua ZHANG, 2001, PROCEEDING ...... STATES OF AMERICA, V98, P6330, DOI	2001	7.51	**2003**	2006	
Latil A, 2001, CANCER RES, V61, P1919	2001	7	**2003**	2005	
Horvath LG, 2001, CANCER RES, V61, P5331	2001	6.5	**2003**	2006	
Fixemer T, 2003, PROSTATE, V54, P79, DOI 10.1002/pros.10171, DOI	2003	5.89	**2004**	2008	
Zhu XG, 2004, AM J PATHOL, V164, P2003, DOI 10.1016/S0002-9440(10)63760-1, DOI	2004	5.91	**2006**	2009	
Guerini V, 2005, CANCER RES, V65, P5445, DOI 10.1158/0008-5472.CAN-04-1941, DOI	2005	5.23	**2007**	2010	
Prins GS, 2008, STEROIDS, V73, P233, DOI 10.1016/j.steroids.2007.10.013, DOI	2008	6.49	**2009**	2013	
Ricke WA, 2008, FASEB J, V22, P1512, DOI 10.1096/fj.07-9526com, DOI	2008	6.49	**2009**	2013	
Mak P, 2010, CANCER CELL, V17, P319, DOI 10.1016/j.ccr.2010.02.030, DOI	2010	9.82	**2011**	2015	
Leung YK, 2010, ENDOCR-RELAT CANCER, V17, P675, DOI 10.1677/ERC-09-0294, DOI	2010	6.38	**2011**	2015	
Jemal A, 2010, CA-CANCER J CLIN, V60, P277, DOI 10.3322/caac.20073, DOI	2010	6	**2011**	2015	
Ellem SJ, 2009, ANN NY ACAD SCI, V1155, P174, DOI 10.1111/j.1749-6632.2009.04360.x, DOI	2009	5.3	**2011**	2014	
Ellem SJ, 2010, J STEROID BIOCHEM, V118, P246, DOI 10.1016/j.jsbmb.2009.10.015, DOI	2010	5.15	**2011**	2013	
Mcpherson SJ, 2010, P NATL ACAD SCI USA, V107, P3123, DOI 10.1073/pnas.0905524107, DOI	2010	7.53	**2012**	2015	
Bonkhoff H, 2009, EUR UROL, V55, P533, DOI 10.1016/j.eururo.2008.10.035, DOI	2009	6.82	**2012**	2014	
Dey P, 2012, MOL ENDOCRINOL, V26, P1991, DOI 10.1210/me.2012.1227, DOI	2012	7.1	**2014**	2017	
Safarinejad MR, 2012, MOL CARCINOGEN, V51, P0, DOI 10.1002/mc.21870, DOI	2012	5.31	**2014**	2017	
Hussain S, 2012, PLOS ONE, V7, P0, DOI 10.1371/journal.pone.0040732, DOI	2012	4.89	**2014**	2016	
Siegel R, 2014, CA-CANCER J CLIN, V64, P9, DOI 10.1136/bmj.g1502, DOI	2014	6.35	**2015**	2018	
Dey P, 2014, ONCOGENE, V33, P4213, DOI 10.1038/onc.2013.384, DOI	2014	4.87	**2015**	2018	
Nelson AW, 2014, ENDOCR-RELAT CANCER, V21, P0, DOI 10.1530/ERC-13-0508, DOI	2014	5.55	**2016**	2018	
Christoforou P, 2014, MOL MED, V20, P427, DOI 10.2119/molmed.2014.00105, DOI	2014	6.43	**2017**	2019	
Bonkhoff H, 2018, PROSTATE, V78, P2, DOI 10.1002/pros.23446, DOI	2018	8.16	**2019**	2022	

Supplementary Figure S5. Top 25 references with the strongest citation bursts.
